# Complications of cranioplasty in relationship to traumatic brain injury: a systematic review and meta-analysis

**DOI:** 10.1007/s10143-021-01511-7

**Published:** 2021-03-08

**Authors:** David Shepetovsky, Gianluca Mezzini, Lorenzo Magrassi

**Affiliations:** 1 Department of Clinical Surgical Diagnostic and Pediatric Sciences, University of Pavia, Viale Brambilla 74, 27100 Pavia, Italy; 2 IRCCS Fondazione Policlinico S. Matteo, Pavia, Italy

**Keywords:** Cranioplasty, Decompressive craniectomy, Traumatic brain injury, Complications

## Abstract

Despite being a common procedure, cranioplasty (CP) is associated with a variety of serious, at times lethal, complications. This study explored the relationship between the initial injury leading to decompressive craniectomy (DC) and the rates and types of complications after subsequent CP. It specifically compared between traumatic brain injury (TBI) patients and patients undergoing CP after DC for other indications.

A comprehensive search of PubMed, Scopus, and the Cochrane Library databases using PRISMA guidelines was performed to include case-control studies, cohorts, and clinical trials reporting complication data for CP after DC. Information about the patients’ characteristics and the rates of overall and specific complications in TBI and non-TBI patients was extracted, summarized, and analyzed.

A total of 59 studies, including the authors’ institutional experience, encompassing 9264 patients (4671 TBI vs. 4593 non-TBI) met the inclusion criteria; this total also included 149 cases from our institutional series. The results of the analysis of the published series are shown both with and without our series 23 studies reported overall complications, 40 reported infections, 10 reported new-onset seizures, 13 reported bone flap resorption (BFR), 5 reported post-CP hydrocephalus, 10 reported intracranial hemorrhage (ICH), and 8 reported extra-axial fluid collections (EFC). TBI was associated with increased odds of BFR (odds ratio [OR] 1.76, *p* < 0.01) and infection (OR 1.38, *p* = 0.02). No difference was detected in the odds of overall complications, seizures, hydrocephalus, ICH, or EFC.

Awareness of increased risks of BFR and infection after CP in TBI patients promotes the implementation of new strategies to prevent these complications especially in this category of patients.

**Supplementary Information:**

The online version contains supplementary material available at 10.1007/s10143-021-01511-7.

## Introduction

Management of severe intracranial hypertension, when conservative therapies fail, may require DC, which is often followed by CP after patient stabilization and optimization [1, 2]. Despite being a time-honored and seemingly “straightforward” procedure, CP is burdened by a complication rate of up to 40%, some of which are severe enough to compromise the outcome of the initial brain injury [3–5]. Documented complications following CP include, among others: seizures, ICH, EFC, infections, hydrocephalus, BFR, mechanical complications (MC), cerebral edema, and neurological deficits (NDs) [3, 6–9].

In recent years, a considerable number of studies explored the various risk factors and pathological mechanisms underlying complications of CP after different brain injuries not limited to trauma. However, very few of those studies evaluated whether the type of initial injury may represent a risk factor for post-CP complications [10–12].

Victims of TBI constitute the largest group of patients requiring DC and subsequent CP [13]. In those patients, factors such as impaired wound sterility due to traumatic contamination, scalp lacerations, multiple complex skull fractures, diffuse axonal injury, and pericontusional ischemia may all increase the risk of post-CP complications and be detrimental to the long-term outcome of the patient [2, 14–16].

This study aimed to compare the rates and types of post-CP complications between TBI patients and patients suffering from other primary pathologies, via a systematic review and meta-analysis of the literature, supplemented by our institutional experience. By elucidating the relationship between TBI and specific complications following CP, it may be possible to prevent neurological damage and minimize the risk of complications linked to CP, thus potentially improving neurologic outcome.

## Materials and methods

### Search strategy

A comprehensive literature search to identify articles of interest was performed in accordance with the PRISMA (Preferred Reporting Items for Systematic Reviews and Meta-Analyses) guidelines [17]. PubMed, Scopus, and the Cochrane Library were searched using the keywords: “cranioplasty,” “decompressive craniectomy,” “complication,” “outcome,” “traumatic brain injury,” and “TBI.” The search included all papers published between January 2000 and May 2020. The reference sections of the identified studies were reviewed to identify any additional relevant articles.

### Study selection

Case-control studies, cohort studies, or clinical trials reporting on the relationship between the indication for DC and the type and rate of related post-CP complications in human adults were included in the analyses, unless they did not include enough data to calculate the number of patients that underwent DC for each indication considered in the study or the number of complications following subsequent CP.

Comments, letters, technical notes, editorials, case reports, or case series relating individually selected cases were excluded. Meta-analyses and reviews were also excluded; however, referenced articles were thoroughly screened for possible inclusion.

Non-English articles were excluded, unless the article was part of a related systematic review. Studies that involved animals, included non-calvarial or maxillofacial procedures, or focused exclusively on the pediatric population were excluded. Studies were excluded if they reported patients who initially underwent craniotomy for reasons other than decompression unless it was possible to separate them from the DC patients.

Table 1 summarizes the inclusion and exclusion criteria.

**Table 1:** Study selection criteria

Inclusion criteria	Exclusion criteria
Case-control studies, cohort studies, or clinical trials, large case series	Comments, letters, technical notes, editorials, meta-analyses, reviews, case reports, or individually selected case series
Includes data on the indication for decompressive craniectomy and the type and rate of related post-cranioplasty complications	Focuses on animals, pediatric patients, or cranioplasties done for reasons other than post-decompressive craniectomy
	Published in a language other than English
	Contains insufficient data for meta-analysis

### Data extraction

The following information was collected from eligible articles: (1) number of patients in the cohort, (2) indication for DC, (3) mean age, (4) male to female ratio, (5) time interval between DC and CP, (6) implant materials used for CP (e.g., preserved autologous bone, methyl methacrylate, and polyether ether ketone), (7) mean post-op follow-up time, and (8) incidence and types of post-CP complications.

Complications were grouped into the following categories: (1) new-onset seizures; (2) intracranial hemorrhage (including intracerebral hemorrhage, subdural hematoma, and epidural hematoma); (3) nonhemorrhagic extra-axial fluid collections (including subgaleal and subdural effusion, cerebrospinal fluid leak or fistula, and hygroma); (4) infection (including surgical site infection, wound healing disturbances and wound dehiscence, graft infection, empyema, brain abscess, and osteomyelitis); (5) hydrocephalus (treated with VPS or conservatively); (6) bone flap resorption (including aseptic bone flap necrosis; diagnosed by clinical exam (softness of the reimplanted flap at palpation and/or flap loosening), imaging (CT scan), or both). In our own series, bone flap resorption was defined as any bone defect superior to 0.5 cm in its largest diameter when a late CT scan (obtained at least 2 months after CP) was compared to postoperative (within 24 h from CP) CT scan; (7) other complications include MC, cerebral edema, and NDs. For studies that reported all cases of complications observed in their cohort (rather than focusing only on specific types of complications), the category “overall complications” was calculated by including all subjects affected by one or more of the above complications.

Not all articles provided information about each item; therefore, comparative analysis was limited by the nature of the source data.

Study quality and risk of bias of individual articles were assessed by the Newcastle-Ottawa Scale (NOS)—a three-category, 9-point scale assessing cohort selection, comparability, and outcome [18]. A higher score indicates higher quality.

### Local series of CP patients

A retrospective review was done to identify all cranioplasties performed on adult patients (older than eighteen at the time of the initial injury) at the neurosurgical unit of Fondazione IRCCS San Matteo (Pavia, Italy), between January 1st 2009 and December 31st 2019. All patients or their legal representative when the patient neurological status did not allow an informed decision signed an informed consent authorizing the use of anonymized clinical data for retrospective analysis and clinical research. Relevant clinical and demographic data were extracted from the patients’ medical records, including age, sex, indication for initial DC, timing of CP after initial DC, CP implant material, postoperative complications, and post-CP follow-up time. All data were anonymized before analysis, and parameters characterizing our series were introduced as a bulk in the meta-analysis.

### Data analysis

The data were analyzed using Review Manager 5.3.5 (The Cochrane Collaboration) [19]. Complications were first categorized by type (e.g., overall complications, infection, and seizure) and then split into two groups: “TBI patients” and “non-TBI patients,” according to the original indication for initial DC. TBI patients included every injury described as “trauma” or “head injury” as well as concussion, contusion, and traumatic/non-spontaneous ICH. Open and closed TBI were considered together. The non-TBI patient group included all other indications such as hemorrhagic and ischemic strokes, spontaneous subarachnoid hemorrhage, infections, and tumors.

The pooled rate of every complication type for each indication group was calculated by summing the number of patients reported to suffer the specific complication in each study and then dividing the by the total number of patients who underwent DC for that indication.

For studies that included both TBI and non-TBI patients, odds ratios (ORs) and 95% confidence intervals (CI) for each complication were calculated by dividing the odds in the “TBI” patient group by the odds in the “non-TBI” patient group. To account for the heterogeneity of the data, the Mantel–Haenszel method with random-effects model was used. The *I*^2^ metric was reported to further quantify heterogeneity. *p* values of 0.05 or less were considered statistically significant.

## Results

**Fig. 1:**
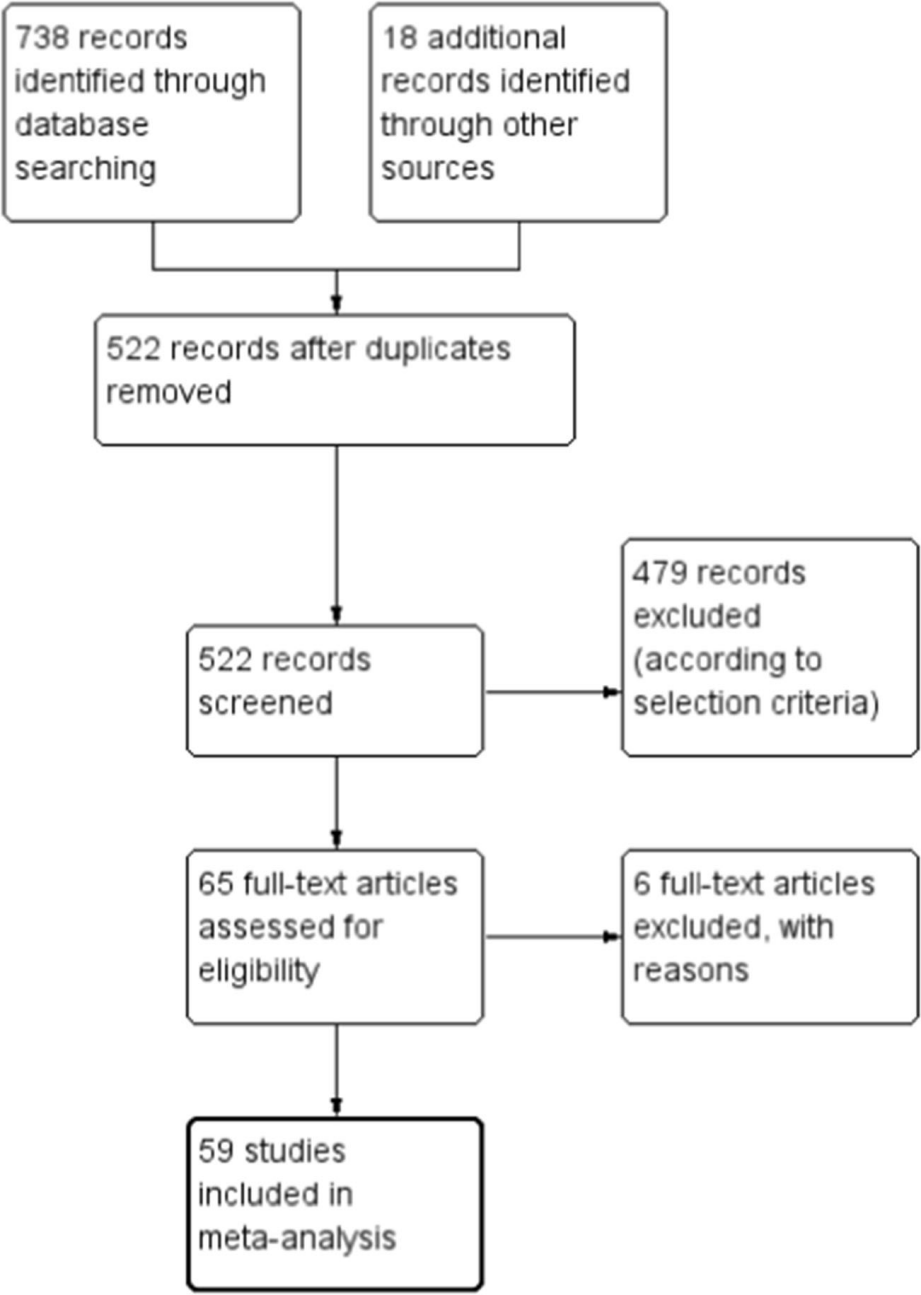
PRISMA flow diagram of literature search strategy and article selection

The initial literature search yielded 738 entries. Eighteen additional articles were found by searching the references of articles. Two-hundred-thirty-four duplicates were found and removed. The remaining 522 studies were screened, and 457 were eliminated based on exclusion criteria. After this, 65 articles were assessed for eligibility and 59 were included in the final analysis (Fig. 1) [10–12, 15, 16, 20–72].

Six articles were excluded for the following reasons: 5 articles utilized the same 2 cohorts [10, 37, 73–75]. Therefore, 1 article for each cohort was selected and the other 3 articles were discarded. One article dealt mostly with pediatric patients (mean age 12.2 years) [76]; 2 articles included a significant proportion (>75%) of cranioplasties done for reasons other than post-DC [77, 78].

Two studies included cranioplasties done both following a DC and for other indications but reported sufficient detail to allow for extraction of the data regarding post-DC cranioplasties only [45, 49].

There was an overlap between 2 studies in the authors, location and time frame in which they were performed [16, 36]. However, the studies focused on different complications (1 reported BFR, and the other dealt with infection, EFC, and IH), and were therefore treated as a single cohort with complication rates pooled from both studies.

Seven of the included studies were published between 2000 and 2009, compared with 52 studies published between 2010 and 2019. This disparity reflects the growing interest for complications of CP as demonstrated by PubMed’s Publication Timeline feature showing an average of 3 ± 3 papers per year during the first decade of the century compared with 35 ± 13 per year during the second decade.

### Study and patient characteristics

The baseline characteristics of the individual studies are summarized in Table 2. Of the studies included, 6 were prospective cohorts and the rest (*n*=53) were retrospective cohorts. Together, the studies comprised a pooled cohort of 9264 patients, out of which 4671 (50%) were affected by TBI. Fifty studies reported mean age, with a pooled mean of 44 ± 1 years. Fifty-six studies reported data about the male to female ratio, with a pooled mean of 65% ± 2% males.

**Table 2:** Characteristics of included studies

Reference	Study design	Number of patients			Mean age (years)	Males (%)	Indications for DC	Described complications	Implant material	Mean DC to CP interval (days)	Follow-up (months)	Quality (NOS)
		Total	TBI	Non-TBI								
Nagayama 2002*	Retrospective cohort	195	28	167	NS	NS	TBI, non-TBI	Infection	ABF	NS	NS	N/A
Shimizu 2002*	Retrospective cohort	70	29	41	NS	NS	TBI, non-TBI	Infection	ABF	NS	NS	N/A
Iwama 2003	Retrospective cohort	49	15	34	51	61	SAH, TBI, VL, ICH, stroke, tumor, infection	Infection, BFR	ABF	NS	59	6★
Moreira-Gonzalez 2003	Retrospective cohort	200	99	101	32	55	TBI, tumor	NS	ABF, PMMA, HA	NS	36	7★
Matsuno 2006	Retrospective cohort	206	64	142	48	60	SAH, ICH, VL, TBI, infection, tumor	Infection	ABF, PMMA, customized	NS	64	7★
Kriegel 2007	Retrospective cohort	39	11	28	40	56	TBI, stroke, ICH, tumor, infection	Infection, BFR, ICH	ABF, PMMA	234	32	6★
Cheng 2008	Retrospective cohort	84	60	24	45	73	TBI, ICH, stroke, ST, VL, tumor, infection	Infection	ABF, PMMA	370	NS	6★
Beauchamp 2010	Retrospective cohort	69	69	0	30^	80	TBI	HC, infection	ABF, synthetic	87^	NS	6★
Chang 2010	Retrospective cohort	212	79	133	44	57	SAH, TBI, stroke, tumor, infection, VL, other	NS	ABF, synthetic	160	NS	7★
Inamasu 2010	Retrospective cohort	70	33	37	46	53	TBI, SAH, ICH, stroke	Infection	ABF	39	50	7★
Stephens 2010	Retrospective cohort	108	108	0	26	99	TBI	Infection, seizure, ICH	PMMA, TM	190	NS	6★
Güresir 2011	Prospective cohort	196	74	122	51	55	TBI, SAH, ICH, stroke, other	Infection, EFC, ICH	ABF	97	>6	8★
Huang 2011	Prospective cohort	135	135	0	43	69	TBI	Infection, ICH, EFC	ABF	83	33	6★
Lee 2011	Retrospective cohort	59	39	20	48	66	TBI, SAH, stroke, tumor	EFC, ICH, infection, BFR	ABF, PMMA	196	11.5	8★
Sobani 2011	Retrospective cohort	96	55	41	33	73	TBI, tumor, infection, stroke	HC, infection, ICH, seizures, EFC, other	ABF, PMMA, customized	90	13	8★
Archavalis 2012	Retrospective cohort	200	51	149	53^	55	SAH, TBI, stroke, ICH, infection	Infection, ICH, EFC, other	ABF	75	43	8★
Im 2012	Retrospective cohort	131	61	70	50	62	VL, TBI, tumor	Infection	ABF, PMMA, PEEK, bone cement	<90	NS	6★
Schuss 2012	Prospective cohort	280	98	182	46	52	TBI, stroke, SAH, ICH	ICH, infection, EFC, BFR	ABF	103	NS	6★
Tantawi 2012	Retrospective cohort	200	200	0	25	100	TBI	Infection, seizure, other	PMMA, TM	NS	29	6★
Bobinski 2013	Retrospective cohort	49	49	0	43	78	TBI	ICH, infection, BFR, other	ABF, PMMA	114	5	6★
Dünisch 2013	Retrospective cohort	372	134	238	49	57	TBI, SAH, stroke, ICH, tumor	BFR	ABF	78^	12	7★
Lee 2013	Retrospective cohort	236	142	94	38	78	TBI, ICH, stroke, infection, tumor	Seizures, infection, ICH, other	TM, ABF, PEEK	NS	>12	8★
Oladunjoye 2013	Retrospective cohort	62	44	18	NS	60	TBI, stroke, ICH, SAH	HC, EFC, infections	ABF	54^	NS	8★
Piedra 2013	Retrospective cohort	74	0	74	47	50	Stroke, ICH, VL	ICH, infection, HC, BFR	ABF, synthetic	91	13	7★
Schuss 2013	Retrospective cohort	254	89	165	45	52	TBI, stroke, SAH, ICH, other	BFR	ABF	103	12	8★
Walcott 2013	Retrospective cohort	239	146	93	42	66	VL, stroke, TBI	ICH, infection, HC, seizures	ABF, synthetic	183	15	8★
Wachter 2013	Retrospective cohort	136	52	84	45	68	TBI, stroke, SAH, ICH, infection, ST, unknown	BFR, infection, ICH, EFC, other	ABF, PMMA	111	NS	7★
Cheng 2014	Prospective cohort	290	199	91	50	67	TBI, stroke, ICH, SAH, tumor, other	Infection	ABF	60	6-12	7★
Klinger 2014	Retrospective cohort	249	118	131	44	63	TBI, SAH, ICH, stroke, infection, tumor, other	Infection, BFR, ICH	ABF, acrylic	NS	NS	7★
Mukherjee 2014	Retrospective cohort	174	69	105	41	60	TBI, tumor, infection	Seizures, ICH, infection, other	TM	311	NS	6★
Schoekler 2014	Retrospective cohort	58	18	40	46	57	Stroke, TBI, ICH, tumor	BFR	ABF, PMMA, PEEK,	233	6	8★
Sundseth 2014	Retrospective cohort	47	0	47	48	57	Stroke, ICH, tumor	Infection, BFR	ABF	97^	41^	6★
Brommeland 2015	Retrospective cohort	71	58	13	31^	62	TBI, stroke	Infection, ICH, BFR, other	ABF, synthetic	74^	10^	7★
Chen 2015	Retrospective cohort	7	7	0	47	71	TBI	Infection	TM	54	29	7★
Paredes 2015	Prospective cohort	55	28	27	43	67	TBI, ICH, VL	Infection, ICH	ABF, PEEK, PMMA	309	NS	5★
Rosseto 2015	Retrospective cohort	45	38	7	32	82	Stroke, infection, TBI, unknown	Infection	ABF, synthetic	NS	2-27	6★
Tsang 2015	Retrospective cohort	162	68	94	49	63	VL, TBI, infection, tumor	Infection, BFR, seizures, other	ABF, acrylic, TM	162	58	8★
Von Der Breile 2015	Retrospective cohort	219	159	60	43	NS	TBI, stroke	Infection, BFR	ABF, PMMA, customized	NS	NS	7★
Borger 2016	Retrospective cohort	75	0	75	52	48	stroke	Infection, ICH, other	NS	145	NS	6★
Chaturvedi 2016	Retrospective cohort	74	74	0	32^	71	TBI	Infection, seizure, ICH, EFC, other	ABF, TM, acrylic	305	32	6★
Daou 2016	Retrospective cohort	114	15	99	51	47	SAH, ICH, stroke, TBI, ICH, VL	BFR, HC, ICH, seizures, infection	ABF	180	25	8★
Honeybul 2016	Retrospective cohort	512	330	182	39	71	TBI, stroke. tumor, infection, SAH, ICH	EFC, ICH, seizures, infection, BFR	ABF, TM, mesenchymal stromal cells	98	NS	7★
Krause-Titz 2016	Retrospective cohort	248	80	168	46	51	TBI, SAH, stroke, tumor	HC, seizures, ICH, infection, other	ABF, PMMA, PMMA+TM, customized	231	NS	7★
Pierson 2016	Retrospective cohort	39	24	15	57	61	TBI, VL, ICH, stroke, infection, tumor	Infection, seizures	ABF, customized	124	NS	7★
Quah 2016	Prospective cohort	70	47	23	40	71	TBI, ICH, stroke, other	Infection	ABF, TM, acrylic, PEEK	NS	23	5★
Riordan 2016	Retrospective cohort	186	92	94	36	62	TBI, SAH, infection, ICH, tumor, stroke	Infection	ABF, synthetic	220	NS	7★
Roberts 2016	Retrospective cohort	14	14	0	24	100	TBI	Infection	ABF, synthetic	NS	NS	5★
Shibahashi 2017	Retrospective cohort	155	82	73	57^	59	TBI, SAH, ICH, stroke	Infection, ICH	ABF, synthetic	44	6	8★
Abode-Iamah 2018	Retrospective cohort	258	139	119	49	62	ICH, TBI, tumor, other	Infection, seizures, HC, BFR, EFC, ICH	ABF, synthetic	NS	NS	7★
Jin 2018	Retrospective cohort	57	36	21	43	70	TBI, stroke, SAH, ICH, tumor	Infection, BFR	ABF	136	43	7★
Morton 2018	Retrospective cohort	754	388	366	44	60	TBI, ICH, SAH, stroke, infection, tumor	HC, BFR, seizures, ICH, other	ABF, synthetic	NS	8	8★
Posti 2018	Retrospective cohort	155	40	115	43	66	TBI, stroke, tumor, infection, ICH, SAH, other	NS	ABF, TM, HA, FRC-BG, PMMA, PEEK, PE	330	12	8★
Kim 2019	Retrospective cohort	126	54	72	51	63	SAH, tumor, stroke, ICH, TBI	Infection, BFR	ABF	NS	NS	6★
Shih 2019	Retrospective cohort	189	113	76	52^	67	TBI, stroke, tumor, infection	Seizures, ICH, infection, other	ABF, PMMA, TM	52	24	8★
Yeap 2019	Retrospective cohort	336	220	116	45	67	ICH, VL, stroke, infection, TBI, tumor	Seizures	ABF, TM, PMMA	312	NS	7★
Alkhaibary 2020	Retrospective cohort	103	78	25	31	84	TBI, stroke, SAH, other	Infection	ABF	115	7	8★
Goedemans 2020	Retrospective cohort	145	27	118	44	46	Stroke, SAH, TBI, ST, ICH, infection, tumor	ICH, infection, EFC, HC	ABF, synthetic	136	12	8★
Rashidi 2020	Retrospective cohort	303	110	193	51	59	TBI, stroke, ICH, SAH, tumor, infection, other	BFR	ABF	182	13	8★
Our series	Retrospective cohort	149	56	93	53	57	TBI, infection, tumor stroke, ICH	Infection, seizure, HC, ICH, BFR	ABF, synthetic	159	77	8★

*TBI* traumatic brain injury, *DC* decompressive craniectomy, *CP* cranioplasty, *NOS* Newcastle-Ottawa Scale, *SAH* subarachnoid hemorrhage, *ST* sinus thrombosis, *VL* venous lesion, *ICH* intracranial hemorrhage, *BFR* bone flap resorption, *EFC* extra-axial fluid collection, *HC* hydrocephalus, *NS* not specified, *ABF* autologous bone flap, *PMMA* polymethylmethacrylate, *TM* titanium mesh, *HA* hydroxyapatite, *FRC-BG* fiber-reinforced composite-bioactive glass, *PEEK* polyetheretherketone, *PE* polyethylene, *N/A* not applicable

*Data extracted from Cheng et al. [31] (original article in Japanese); ^ median

Fifty-five studies included CP performed using autologous bone flaps (ABF), with 36 studies also including synthetic materials such as titanium mesh, hydroxyapatite (HA), polymethyl methacrylate (PMMA), polyether-ether-ketone (PEEK), and individually customized implants (containing a combination of the previously mentioned materials). Three studies included only CP performed with synthetic materials.

Time interval from DC to CP ranged between 39 and 370 days, with a median of 196 and a mean of 161 ± 37.5 days. Fourteen studies, including our own series, stratified the time interval according to the initial indication for DC [16, 22, 24, 25, 29, 30, 36, 37, 45, 53, 55, 56, 67]. The pooled mean interval from those studies was 131 ± 62 days for TBI patients, compared with 116 ± 58 days for non-TBI patients. The timing of CP did not significantly differ between the underlying pathologies (difference = 15 days, 95% CI = −37 < diff < 67, and *p* = 0.57).

Study quality ranged from 5 to 8 out of 9 on the Newcastle-Ottawa Scale. Points were deduced mainly for lack of matched cohorts, neglect to mention length of follow-up and lack of representativeness of the exposed cohort of the overall population. Most (*n* = 56) had adequate ascertainment of exposure, sufficient comparability, and good assessment of outcome.

### Institutional experience

The general characteristics of our cohort are summarized in Table 2. It included a total of 149 patients, out of which 56 (37.6%) were initially diagnosed with TBI. The overall complication rate of CP was calculated by considering all complications independently of whether they required treatment or not and added up to 42.3%. There was no statistically significant difference in odds of overall complications between TBI patients (37.5%) and non-TBI patients (45.2%); OR = 0.83 (*p*= 0.56 and CI 0.45–1.54).

The rates of individual complications in our institution were 0.7% for new-onset seizures, 7.4% for ICH, 22.1% for infection, 8.7% for hydrocephalus, and 17.1% for BFR. When subdivided according to complication type, no single complication had significantly different odds in TBI patients compared with non-TBI ones.

### Overall complications

In total, 23 studies reported the overall rates of post-CP complications seen in their cohorts. These included seizures (*n*=5), ICH (*n*=13), EFC (*n*=7), infection (*n*=19 studies), hydrocephalus (*n*=5), BFR (*n*=8), and other complications (*n*=6). Three studies did not specify which complications were considered.

The pooled overall complication rate of CP was 24.6% (683/2779) when considering all indications together, consisting of 27.8% (353/1269) in TBI patients and 21.9% (330/1510) in non-TBI patients.

**Fig. 2:**
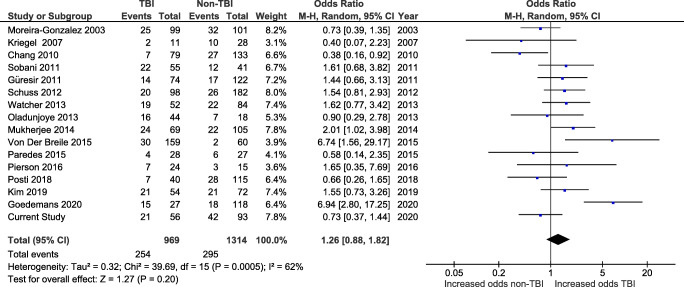
Forest plot of studies reporting overall cranioplasty complications in TBI and non-TBI patients

Sixteen of the studies reporting overall complication rates allowed for comparison between the TBI and non-TBI patients (Fig. 2) (while the other 7 only reported complication rates for a single primary indication). The pooled rate of overall complications from these studies was 24.0% (*n* = 549/2283), ranging from 14.6 to 42.3%.

No significant difference in the odds of overall complications was found in TBI patients (*n* = 254/969 procedures, 26.2%) when compared with non-TBI patients (*n* = 295/1314, 22.5%; OR 1.26, CI 0.88–1.82, and *p* = 0.20) using a random-effects model (*I*^2^ = 62% and *p* = 0.0005). Similar results were obtained when the analysis was conducted without adding our series to the published data (Supplementary Fig. S2).

### Seizures

In total, 10 studies reported new-onset seizure rates. The pooled rate was 13.2% (307/2333). When divided according to initial indication, the rate of seizure was 12.6% (177/1405) in TBI patients and 14.0% (130/928) in non-TBI patients.

**Fig. 3:**
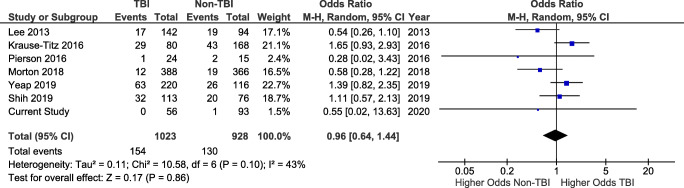
Forest plot of studies reporting new-onset post-cranioplasty seizures in TBI and non-TBI patients

Seven of the studies reporting seizure rates allowed for comparison between TBI and non-TBI patients (Fig. 3). The pooled rate of seizures from these studies was 14.6% (*n* = 284/1951), ranging from 0.7 to 29.0%.

There was no difference in the odds of seizure in TBI patients (*n* = 154/1023 procedures, 15.1%) when compared with non-TBI patients (*n* = 130/928, 14.0%; OR 0.96, CI 0.64–1.44, and *p* = 0.86) using a random-effects model (*I*^2^ = 43% and *p* = 0.10). This was true with (Fig. 3) or without the addition of the cases of our series (Supplementary Fig. S3).

### Intracranial hemorrhage

In total, 10 studies reported ICH rates. The pooled rate was 4.8% (63/1320), 4.6% (28/615) in TBI patients, and 5.0% (35/705) in non-TBI patients.

**Fig. 4:**
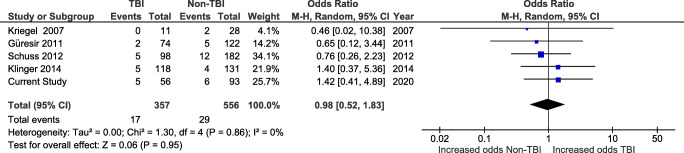
Forest plot of studies reporting post-cranioplasty intracranial hemorrhage in TBI and non-TBI patients

Five of the studies reporting ICH allowed for comparison between TBI and non-TBI patients (Fig. 4). One study reported postoperative epidural hematomas only, while the others, including ours, considered both epidural and subdural hematomas requiring evacuation. One study also considered subgaleal hematomas.

The pooled rate of hemorrhage from these studies was 5.0% (*n* = 46/913), ranging from 3.6 to 7.4%.

There was no difference in the odds of hemorrhage in TBI patients (*n* = 17/357 procedures, 4.8%) when compared with non-TBI patients (*n* = 29/556, 5.2%; OR 0.98, CI 0.52–1.83, and *p* = 0.95) using a random-effects model (*I*^2^ = 0% and *p* = 0.86). This was true with (Fig. 4) or without the addition of the cases of our series (Supplementary Fig. S4).

### Extra-axial fluid collections

In total, 8 studies reported the rates of noninfectious and nonhemorrhagic EFCs. The pooled rate was 5.5% (57/1028), 7.3% (42/578) in TBI patients, and 3.3% (15/450) in non-TBI patients.

**Fig. 5:**
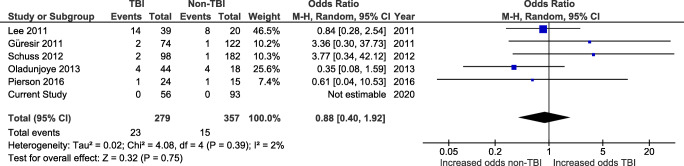
Forest plot of studies reporting post-cranioplasty extra-axial fluid collections in TBI and non-TBI patients

Five of the studies reporting EFC were allowed for comparison between the TBI and non-TBI group (Fig. 5). These included epidural and subdural fluid collections (*n*=3 studies), hygroma (*n*=3), and CSF fistula (*n*=2). In our cohort, no patient suffered from EFC after CP.

The pooled rate of EFC from these studies was 6.0% (*n* = 38/636), ranging from 1.1 to 37.3%.

There was no difference in the odds of EFC in TBI patients (*n* = 23/279 procedures, 8.2%) when compared with non-TBI patients (*n* = 15/357, 4.2%; OR 0.88, CI 0.40–1.92, and *p* = 0.75) using a random-effects model (*I*^2^ = 2% and *p* = 0.39). This was true with (Fig. 5) or without the addition of the cases of our series (Supplementary Fig. S5).

### Infection

In total, 40 studies reported infection rates. The pooled rate was 10.0% (546/5461), consisting of 11.0% (325/2960) in TBI patients and 8.8% (221/2501) in non-TBI patients. In our series, we considered as complicated by infection every cranioplasty associated with persistent fever, raised inflammatory parameters, and/or local swelling or pus. We also considered infected the skull flaps that resulted positive for microbial growth at the time of revision surgery. If both BFR and infection were present in the same patient, the patient was considered as affected by two complications.

**Fig. 6:**
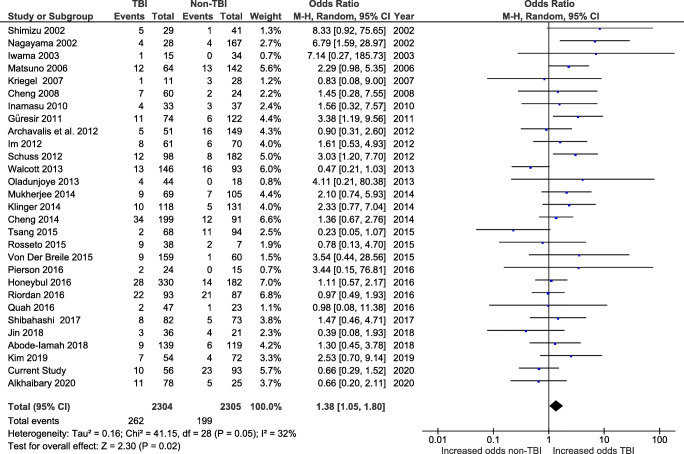
Forest plot of studies reporting post-cranioplasty infection in TBI and non-TBI patients

Twenty-nine of the studies reporting infection rates allowed for comparison between TBI and non-TBI patients (Fig. 6). The pooled rate of infection from these studies was 10.0% (*n* = 461/4609), ranging from 2.0 to 24.4%.

There was a significant increase in the odds of infection in TBI patients (*n* = 262/2304 procedures, 11.4%) when compared with non-TBI patients (*n* = 199/2305, 8.6%; OR 1.38, CI 1.05–1.80, and *p* = 0.02) using a random-effects model (*I*^2^ = 32% and *p* = 0.05). This was true with (Fig. 6) or without the addition of the cases of our series (Supplementary Fig. S6).

### Hydrocephalus post-CP

In total, 5 studies reported rates of hydrocephalus developing after CP. The pooled rate was 12.0% (155/1287), 8.1% (46/568) in TBI patients, and 15.2% (109/719) in non-TBI patients.

**Fig. 7:**
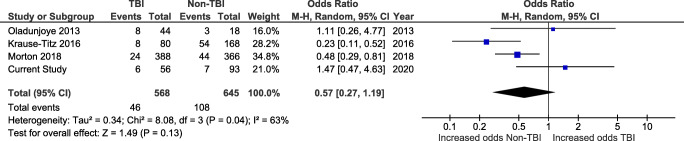
Forest plot of studies reporting post-cranioplasty hydrocephalus in TBI and non-TBI patients

Four of the studies reporting post-CP hydrocephalus allowed for comparison between TBI and non-TBI patients (Fig. 7). Three studies, including ours, defined hydrocephalus as requiring the placement of a ventriculoperitoneal shunt, while one considered both cases that required VPS and those that resolved spontaneously.

The pooled rate of hydrocephalus from these studies was 12.7% (*n* = 154/1213), ranging from 8.7 to 25.0%.

There was no difference in the odds of hydrocephalus in TBI patients (*n* = 46/568 procedures, 8.1%) when compared with non-TBI patients (*n* = 108/645, 16.7%; OR 0.57, CI 0.27–1.19, and *p* = 0.13) using a random-effects model (*I*^2^ = 63% and *p* = 0.04), when cases collected from the literature and our own were considered together (Fig. 7). However, when published cases were analyzed separately, the odds of hydrocephalus in TBI patients were 7.8% (*n*=40/152), compared with 18.3% (*n*=101/552) in non-TBI patients, giving an odds ratio of 0.43 (CI 0.22–0.86). These results were significant (*p* = 0.02) (Supplementary Fig. S7).

### Bone flap resorption

In total, 13 studies reported BFR rates. For calculation purposes, studies that included synthetic implants in their cohorts were considered only if it was possible to extract the number of patients who underwent CP using autologous bone graft. Resorption was determined either by clinical exam (softness of the reimplanted flap at palpation and or flap loosening) or imaging (CT scan) or both. In our own series, bone flap resorption was defined as any bone defect superior to 0.5 cm in its largest diameter when a late CT scan (obtained at least 2 months after CP) was compared to an early (within 24 h) post-CP CT scan. The pooled rate was 14.2% (271/1913). When divided according to initial indication, the rate of BFR was 19.7% (165/837) in TBI patients and 9.8% (106/1082) in non-TBI patients.

**Fig. 8:**
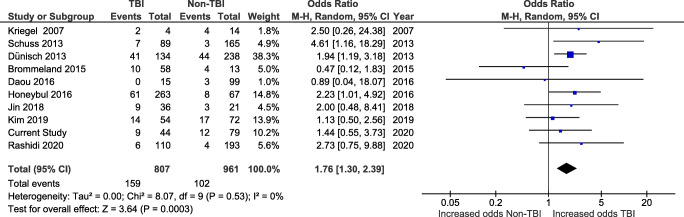
Forest plot of studies reporting post-cranioplasty bone flap resorption in TBI and non-TBI patients

Ten of the studies reporting BFR rates allowed for comparison between TBI and non-TBI patients (Fig. 8). The pooled rate of BFR was 14.8% (*n* = 261/1768), ranging from 3.9 to 33.3%. There was a significant increase in the odds of resorption in TBI patients (*n* = 159/807 procedures, 19.7%) when compared with non-TBI patients (*n* = 102/961, 10.6%; OR 1.76, CI 1.30–2.39, and *p* = 0.0003) using a random-effects model (*I*^2^ = 0% and *p* = 0.53). This was true with (Fig. 8) or without the addition of the cases of our series (Supplementary Fig. S8).

### Other complications

Ten studies reported various MCs, which included contour abnormalities/poor cosmesis (*n*=3 studies) [51, 69, 71], implant displacement (*n*=6) [24, 25, 27, 47, 70, 71], implant loosening (*n*=2) [44, 64], and implant fracture (*n*=1) [44]. The pooled rate of MCs was 3.1%, ranging from 0.4 to 10.0%. Cerebral edema was reported in 2 studies [22, 51], with a pooled rate of 1.07%, ranging from 1.0 to 1.1%. Five studies reported NDs, other than seizures, following CP, including reduced consciousness levels (*n*=1) [70], urinary retention (*n*=1) [51], cerebral contusion (*n*=1) [71], new transient NDs (*n*=1) [66], and worsening of previous NDs (*n*=1) [29]. The pooled rate of NDs was 1.9%, ranging from 0.6 to 6.8%. The reported data for these complications were insufficient for comparison between TBI and non-TBI patients.

## Discussion

This study found no difference in overall complication rates between TBI and non-TBI patients. The heterogeneity of the data was high, indicating that there is a large disagreement between the different studies. This can be explained by several considerations.

First, as can be seen from the table summarizing the study characteristics of the different cohorts (Table 2), the definition of “complications” is quite broad. Some studies measured only complications requiring new surgery [26, 54], while others, as we did in our series, also considered those that could be treated conservatively [28, 35, 42, 51, 56, 57, 62, 79]. Some studies included such outcomes as poor cosmesis [51, 71] or death [56] in their count of complications, while others did not.

Second, comorbidities, choice of graft material, timing of CP, etc. all influence the measured complication rates in a cohort [42, 47, 50, 72]. Since the comparison was made between studies done in different locations, at different times, and on different cohorts, these factors inevitably affected the calculated complication rates.

Third, as can be seen from the subgroup analysis of individual complications, TBI might be a risk factor for some complications, such as infections and bone flap resorption, and not for others, such as hydrocephalus developing post-CP. Thus, when grouping the complications together, the different effects tended to cancel each other.

On the other hand, when individual complications were analyzed, we found that TBI increased the odds of the following specific complications: infection and autologous bone flap resorption. This was true both with and without our series included in the data (Supplementary Fig. S6 and S8). Conversely, the risk of post-CP hydrocephalus resulted significantly higher in non-TBI patients when only published data were analyzed (Supplementary Fig. S7), but not when we added our data (Fig. 7). A possible explanation is that, compared to other complications, only three studies [44, 50, 53] report data on the incidence of hydrocephalus after CP. This relative scarcity of data enhances sensitivity of the statistical analysis to the addition of our series, suggesting that more data will be necessary to clarify the issue.

Our finding of increased odds of autologous bone flap resorption in TBI patients compared to non-TBI patients is in agreement with previous observations based on a smaller number of patients [16, 80]. One explanation is that severe trauma is associated with multiple fractures within the reimplanted autologous bone [16]. The quantity and complexity of the fractures may interfere with the osteointegration of the graft and eventually lead to its resorption [81, 82]. Moreover, bone defects resulting from DC after TBI tend to be larger than those resulting from DC performed for other reasons and may therefore hinder the reintegration of the autologous bone flap after CP [57]. Of note, 12 out of the 13 studies examined in the meta-analysis for BFR (representing 96% of the comparison population) utilized exclusively, as we did, the cryopreservation method to preserve the flaps before CP. Therefore, while it is possible that the method of autologous flap preservation may influence the rates of BFR, this factor was controlled for in our meta-analysis.

Interestingly, our finding in adults is also confirmed in the pediatric population, where patients who undergo CP with autologous bone flap replacement after DC for TBI have been shown to experience even higher rates of BFR, reaching up to 50.0% of cases [83].

The association between DC for TBI and higher post-CP infection rates is also supported by several published studies [23, 51, 67, 84]. Plausible risk factors predisposing TBI patients for infection may include initial contamination of the skull through traumatic discontinuities of the overlying skin and galea or the presence of multiple scars of traumatic and surgical origin which compromise the vascularization of the surgical flap, resulting in delayed wound healing after CP [62, 85]. Here as well, the larger skull defects following DC after TBI and their association with delayed integration of the reimplanted bone flap may also play a role [57]. Lastly, the possible involvement of the air cavities of the skull, involved by fractures or by the decompressive flap, may also increase the infection rate of the reimplanted bone after CP [43].

Our meta-analysis, including our own patient cohort, did not find significant differences in the odds of hydrocephalus, ICH, insurgence of EFCs, or seizures after CP in TBI patients compared with patients undergoing the same surgery for non-TBI pathologies. Hydrocephalus after CP has been associated with nontraumatic SAH [44, 50], and the incidence of ICH after CP is similar in patients with different initial neurologic insults [50]. The pooled rate of EFC in the present analysis was 5.5% (7.3% in TBI versus 3.3% in non-TBI). Kurland et al. reported similar results, with a rate of 5.8% for subdural effusions/hygroma and 6.8% for CSF leaks/fistulas, for an overall rate of 6.1% [86].

One retrospective study showed a significant reduction in odds of overall complication rates in TBI patients [28]. The authors attributed their results to the fact that TBI patients in their cohort were significantly younger on average than patients with other primary pathologies. Although patients affected by TBI were younger than non-TBI patients in our study as well, we did not find a significant negative correlation between TBI and odds of post-CP hydrocephalus.

Out of the studies reporting infection, 19 performed regression analysis to compare between autologous bone and synthetic materials for difference in infection rates. Out of these, only one [48] found significantly lower rates of infection in titanium mesh implants when compared to autologous bone. All the rest found no statistical difference in infection rates. This was confirmed recently by a large meta-analysis [87]. However, most of studies used for the meta-analysis did not specify which type of material was used for each patient. It was therefore impossible for us to determine the ratio of autologous cranioplasties between the TBI and non-TBI patients. In our own series, there was no significant difference in the ratio of cranioplasty using autologous bone flap between the TBI group and the non-TBI group (*χ*^2^, 0.979; DF, 1; *p* < 0.3223).

Finally, we did not find any significant difference in the timing of cranioplasty between TBI and non-TBI patients. Thus, the DC to CP interval does not appear to have influenced the results of our analysis. This conclusion is, in fact, supported both by our own experience and by that of others [62].

## Strengths and limitations

To the best of our knowledge, this is the only meta-analysis to date exploring the differences in both overall and seven specific post-CP complications between TBI patients and other primary pathologies. Kurland et al. had previously considered the rates of post-CP complications according to different DC indications, including trauma, but their study was only a systematic review, utilizing data pooled from different reports to produce a single large cohort [86]. The current study compared between the different cohorts, giving more weight to larger, less biased ones, and attempting to account for the heterogeneity of data using statistical models. In addition, our study is more specific in its categorization of complications into subgroups and analyzed some complications, like seizures, that were not accounted for by Kurland et al.

However, there are several limitations to our analysis. First, the definition of “craniectomy” varied across studies. While studies in which a significant portion of patients underwent craniectomies for purposes other than the reduction of intracranial pressure (e.g., removal of a skull tumor) were excluded, in few cases, it was impossible to quantify the percentage of non-decompressive craniectomies as the reason for craniectomy was not explicitly stated in the paper [32, 48].

Second, as previously stated, the complications controlled for by the individual studies varied significantly. In addition, the definitions for some of the complications differed between the authors, particularly for infection. Some studies only considered infection a complication if it required reoperation [52], whereas others, such as ours, also included infections treated conservatively. Some studies distinguished between surgical site infections, graft infections, and abscesses [66], while others grouped all under the general definition of infection [56]. Cases of cellulitis, meningitis, osteomyelitis, intracranial abscess, and empyema were explicitly specified in some cases studies but not in others [26, 66]. Our analysis therefore grouped all types of infection under a single category.

Third, our analysis did not stratify the data by age, sex, severity of injury, comorbidities, or other variables that may have influenced the results [88]. Neither did it account for the anatomic heterogeneity of CP, as patients with unilateral, bilateral, and bifrontal craniectomies of various sizes were pooled together, even though bifrontal procedures may have significantly higher infection rates and increased risk for reoperation [89].

Fourth, the analysis treated each case of complication as an independent event. This may not be completely accurate, as in reality once a complication has accrued, it tends to predispose the patient for other complications [88].

Lastly, the analysis was susceptible to publication bias, as most of the data were derived from published articles, even though we integrated data from the literature with our direct surgical experience and performed separate analysis with and without the addition of our series. Overall results of the analysis of the two sets of data were largely in agreement, with the exception of post-CP hydrocephalus, as already discussed above.

Nonetheless, the idea behind this study was to try to shed light on the ongoing debate about whether or not TBI as primary pathology influences the rates of post-CP complications. The heterogeneity of the cohorts and data used for the analysis reflects the sacrifice of some of its validity in favor of generalizability, mirroring actual clinical practice, where the spectrum of CP patients, material, surgical techniques, and complications is wide and varied.

## Conclusion

DC is an effective means to control ICP and mitigate the risk of herniation in patients with cerebral edema resulting from a wide range of conditions, and subsequent CP is crucial to restore cranial integrity and prevent SSFS [2]. However, while those procedures are technically straightforward, they place the patient at risk for many nontrivial complications, which can negatively impact outcome [86].

Our systematic review and meta-analysis investigated the difference in complication rates after CP following DC, for TBI and all other indications considered together. Our results suggest that there is no significant difference in the overall complication rates between the two groups. However, when we analyzed specific complications separately, we found a significant increase in odds of autologous bone flap resorption and of infection in TBI patients. We hope that our findings will increase the awareness to these specific complications and promote the development of new strategies to decrease the risk of such complications, which may significantly compromise the already difficult pathway to recovery of trauma patients, even at advanced stages of healing.

Furthermore, as numbers of DC and CP continue to rise [90], it will become increasingly important to be aware of the actual risk of complications encountered by specific patient populations. Neurosurgeons, neurologists, and patients will need to know the risks of the procedure to be able to make informed decisions and develop new protocols to prevent those complications.

## Supplementary information

ESM 1 (PNG 584 kb)

High resolution image (EPS 33685 kb)

ESM 2 (PNG 415 kb)

High resolution image (EPS 33144 kb)

ESM 3 (PNG 372 kb)

High resolution image (EPS 33023 kb)

ESM 4 (PNG 390 kb)

High resolution image (EPS 33116 kb)

ESM 5 (PNG 1639 kb)

High resolution image (EPS 2709 kb)

ESM 6 (PNG 370 kb)

High resolution image (EPS 33142 kb)

ESM 7 (PNG 489 kb)

High resolution image (EPS 33568 kb)

## Data Availability

All relevant data are contained in the manuscript and supplementary materials.

## References

[1] Hutchinson PJ, Kolias AG, Tajsic T, Adeleye A, Aklilu AT, Apriawan T, Bajamal AH, Barthélemy EJ, Devi BI, Bhat D, Bulters D, Chesnut R, Citerio G, Cooper DJ, Czosnyka M, Edem I, El-Ghandour NMF, Figaji A, Fountas KN, Gallagher C, Hawryluk GWJ, Iaccarino C, Joseph M, Khan T, Laeke T, Levchenko O, Liu B, Liu W, Maas A, Manley GT, Manson P, Mazzeo AT, Menon DK, Michael DB, Muehlschlegel S, Okonkwo DO, Park KB, Rosenfeld JV, Rosseau G, Rubiano AM, Shabani HK, Stocchetti N, Timmons SD, Timofeev I, Uff C, Ullman JS, Valadka A, Waran V, Wells A, Wilson MH, Servadei F (2019). Consensus statement from the International Consensus Meeting on the Role of Decompressive Craniectomy in the Management of Traumatic Brain Injury: consensus statement. Acta Neurochir (Wien).

[2] Kolias AG, Kirkpatrick PJ, Hutchinson PJ (2013). Decompressive craniectomy: past, present and future. Nat Rev Neurol.

[3] Acciarri N, Nicolini F, Martinoni M (2016). Cranioplasty: routine surgical procedure or risky operation?. World J Surg Res.

[4] Sahoo NK, Tomar K, Thakral A, Rangan NM (2018). Complications of cranioplasty. J Craniofac Surg.

[5] Sviri GE (2015). Massive cerebral swelling immediately after cranioplasty, a fatal and unpredictable complication: report of 4 cases. J Neurosurg.

[6] Ausman JI, Andrabi SM, Sarmast AH, Kirmani AR, Bhat AR (2017). Cranioplasty: indications, procedures, and outcome-an institutional experience. Surg Neurol Int.

[7] Killeen T, Fortunati M, Myanger E, Rüfenacht D, Ryskeldiyev N, Akshulakov S, Cesnulis E (2019). Symptomatic tension pneumocephalus following Palacos® cranioplasty in a shunted patient. Br J Neurosurg.

[8] Lang SS, Grady MS, Quinones-Hinojosa A (2012). Surgical management of major skull defects and potential complications. Schmidek and Sweet Operative Neurosurgical Techniques: Indications, Methods, and Results.

[9] Qiu S, You W, Wang H, Zhou X, Yang X (2019). Allergic epidural effusion following polyetheretherketone cranioplasty. J Craniofac Surg.

[10] Honeybul S, Ho KM (2016). Cranioplasty: morbidity and failure. Br J Neurosurg.

[11] Rosseto RS, Giannetti AV, De Souza Filho LD, Faleiro RM (2015). Risk factors for graft infection after cranioplasty in patients with large hemicranial bony defects. World Neurosurg.

[12] Walcott BP, Kwon CS, Sheth SA, Fehnel CR, Koffie RM, Asaad WF, Nahed BV, Coumans JV (2013). Predictors of cranioplasty complications in stroke and trauma patients. J Neurosurg.

[13] Kolias AG, Viaroli E, Rubiano AM, Adams H, Khan T, Gupta D, Adeleye A, Iaccarino C, Servadei F, Devi BI, Hutchinson PJ (2018). The current status of decompressive craniectomy in traumatic brain injury. Curr Trauma Reports.

[14] Khan AA, Banerjee A (2010). The role of prophylactic anticonvulsants in moderate to severe head injury. Int J Emerg Med.

[15] Riordan MA, Simpson VM, Hall WA (2016). Analysis of factors contributing to infections after cranioplasty: a single-institution retrospective chart review. World Neurosurg.

[16] Schuss P, Vatter H, Oszvald A, Marquardt G, Imöhl L, Seifert V, Güresir E, Oszvald Á, Marquardt G, Imöhl L, Seifert V, Güresir E (2013). Bone flap resorption: risk factors for the development of a long-term complication following cranioplasty after decompressive craniectomy. J Neurotrauma.

[17] Moher D, Liberati A, Tetzlaff J, Altman DG, Altman D, Antes G, Atkins D, Barbour V, Barrowman N, Berlin JA, Clark J, Clarke M, Cook D, D’Amico R, Deeks JJ, Devereaux PJ, Dickersin K, Egger M, Ernst E, Gøtzsche PC, Grimshaw J, Guyatt G, Higgins J, Ioannidis JPA, Kleijnen J, Lang T, Magrini N, McNamee D, Moja L, Mulrow C, Napoli M, Oxman A, Pham B, Rennie D, Sampson M, Schulz KF, Shekelle PG, Tovey D, Tugwell P (2009) Preferred reporting items for systematic reviews and meta-analyses: the PRISMA statement. PLoS Med:6. 10.1371/journal.pmed.1000097 10.1371/journal.pmed.1000097 PMC2707599 19621072

[18] Wells G, Shea B, O’Connell D, Peterson J, Welch V, Losos M, Tugwell P (2006). The Newcastle-Ottawa Scale (NOS) for assessing the quality of nonrandomised studies in meta-analyses.

[19] RevMan | Cochrane Training. https://training.cochrane.org/online-learning/core-software-cochrane-reviews/revman. Accessed 1 Jun 2020

[20] Abode-Iyamah KO, Chiang HY, Winslow N, Park B, Zanaty M, Dlouhy BJ, Flouty OE, Rasmussen ZD, Herwaldt LA, Greenlee JD (2018). Risk factors for surgical site infections and assessment of vancomycin powder as a preventive measure in patients undergoing first-time cranioplasty. J Neurosurg.

[21] Alkhaibary A, Alharbi A, Abbas M, Algarni A, Abdullah JM, Almadani WH, Khairy I, Alkhani A, Aloraidi A, Khairy S (2020). Predictors of surgical site infection in autologous cranioplasty: a retrospective analysis of subcutaneously preserved bone flaps in abdominal pockets. World Neurosurg.

[22] Archavlis E, Carvi Y, Nievas M (2012). The impact of timing of cranioplasty in patients with large cranial defects after decompressive hemicraniectomy. Acta Neurochir (Wien).

[23] Beauchamp KM, Kashuk J, Moore EE, Bolles G, Rabb C, Seinfeld J, Szentirmai O, Sauaia A (2010). Cranioplasty after postinjury decompressive craniectomy: is timing of the essence?. J Trauma.

[24] Bobinski L, Koskinen L-OD, Lindvall P (2013). Complications following cranioplasty using autologous bone or polymethylmethacrylate - retrospective experience from a single center. Clin Neurol Neurosurg.

[25] Borger V, Schuss P, Kinfe TMTMTM, Vatter H, Güresir E (2016). Decompressive craniectomy for stroke: early cranioplasty is a predictor for postoperative complications. World Neurosurg.

[26] Von Der Brelie C, Stojanovski I, Meier U, Lemcke J (2015). Open traumatic brain injury is a strong predictor for aseptic bone necrosis after cranioplasty surgery: a retrospective analysis of 219 patients. J Neurol Surgery, Part A Cent Eur Neurosurg.

[27] Brommeland T, Rydning PN, Pripp AH, Helseth E (2015) Cranioplasty complications and risk factors associated with bone flap resorption. Scand J Trauma Resusc Emerg Med:23. 10.1186/s13049-015-0155-6 10.1186/s13049-015-0155-6 PMC4595108 26437934

[28] Chang V, Hartzfeld P, Langlois M, Mahmood A, Seyfried D (2010). Outcomes of cranial repair after craniectomy: Clinical article. J Neurosurg.

[29] Chaturvedi J, Botta R, Prabhuraj AR, Shukla D, Bhat DI, Indira Devi B (2016). Complications of cranioplasty after decompressive craniectomy for traumatic brain injury. Br J Neurosurg.

[30] Chen S-T, Chang C-J, Su W-C, Chang L-W, Chu I-H, Lin M-S (2015). 3-D titanium mesh reconstruction of defective skull after frontal craniectomy in traumatic brain injury. Injury.

[31] Cheng C-HCH, Lee HCH-C, Chen CCC-C, Cho DYD-Y, Lin HLH-L (2014). Cryopreservation versus subcutaneous preservation of autologous bone flaps for cranioplasty: comparison of the surgical site infection and bone resorption rates. Clin Neurol Neurosurg.

[32] Cheng YK, Weng HH, Yang JT, Lee MH, Wang TC, Chang CN (2008). Factors affecting graft infection after cranioplasty. J Clin Neurosci.

[33] Daou B, Zanaty M, Chalouhi N, Dalyai R, Jabbour P, Yang S, Rosenwasser RH, Tjoumakaris S (2016). Low incidence of bone flap resorption after native bone cranioplasty in adults. World Neurosurg.

[34] Dünisch P, Walter J, Sakr Y, Kalff R, Waschke A, Ewald C (2013). Risk factors of aseptic bone resorption: a study after autologous bone flap reinsertion due to decompressive craniotomy - Clinical article. J Neurosurg.

[35] Goedemans T, Verbaan D, van der Veer O, Bot M, Post R, Hoogmoed J, Lequin MB, Buis DR, Vandertop WP, Coert BA, van den Munckhof P (2020). Complications in cranioplasty after decompressive craniectomy: timing of the intervention. J Neurol.

[36] Güresir E, Vatter H, Schuss P, Oszvald Á, Raabe A, Seifert V, Beck J (2011). Rapid closure technique in decompressive craniectomy. Clinical article. J Neurosurg.

[37] Huang Y-HH, Lee T-CC, Chen W-FF, Wang Y-MM (2011). Safety of the nonabsorbable dural substitute in decompressive craniectomy for severe traumatic brain injury. J Trauma.

[38] Im SH, Jang DK, Han YM, Kim JT, Chung DS, Park YS (2012). Long-term incidence and predicting factors of cranioplasty infection after decompressive craniectomy. J Korean Neurosurg Soc.

[39] Inamasu J, Kuramae T, Nakatsukasa M (2010). Does difference in the storage method of bone flaps after decompressive craniectomy affect the incidence of surgical site infection after cranioplasty? Comparison between subcutaneous pocket and cryopreservation. J Trauma.

[40] Iwama T, Yamada J, Imai S, Shinoda J, Funakoshi T, Sakai N, Sekhar LN, Stimac D, Sindou MP, Haines SJ, Kawase T (2003). The use of frozen autogenous bone flaps in delayed cranioplasty revisited. Neurosurgery.

[41] Jin SW, Kim SD, Ha SK, Lim DJ, Lee H, You HJ (2018). Analysis of the factors affecting surgical site infection and bone flap resorption after cranioplasty with autologous cryopreserved bone: the importance of temporalis muscle preservation. Turk Neurosurg.

[42] Kim JH, Hwang SY, Kwon TH, Chong K, Yoon WK, Kim JH (2019). Defining “early” cranioplasty to achieve lower complication rates of bone flap failure: resorption and infection. Acta Neurochir (Wien).

[43] Klinger DR, Madden C, Beshay J, White J, Gambrell K, Rickert K (2014). Autologous and acrylic cranioplasty: a review of 10 years and 258 cases. World Neurosurg..

[44] Krause-Titz UR, Warneke N, Freitag-Wolf S, Barth H, Mehdorn HM (2016). Factors influencing the outcome (GOS) in reconstructive cranioplasty. Neurosurg Rev.

[45] Kriegel RJ, Schaller C, Clusmann H (2007). Cranioplasty for large skull defects with PMMA (polymethylmethacrylate) or tutoplast® processed autogenic bone grafts. Zentralbl Neurochir.

[46] Lee JW, Kim JH, Kang HI, Moon BG, Lee SJ, Kim JS (2011). Epidural fluid collection after cranioplasty: fate and predictive factors. J Korean Neurosurg Soc.

[47] Lee L, Ker J, Quah BL, Chou N, Choy D, Yeo TT (2013). A retrospective analysis and review of an institution’s experience with the complications of cranioplasty. Br J Neurosurg.

[48] Matsuno A, Tanaka H, Iwamuro H, Takanashi S, Miyawaki S, Nakashima M, Nakaguchi H, Nagashima T (2006). Analyses of the factors influencing bone graft infection after delayed cranioplasty. Acta Neurochir (Wien).

[49] Moreira-Gonzalez A, Jackson IT, Miyawaki T, Barakat K, DiNick V (2003). Clinical outcome in cranioplasty: critical review in long-term follow-up. J Craniofac Surg..

[50] Morton RP, Abecassis IJ, Hanson JF, Barber JK, Chen M, Kelly CM, Nerva JD, Emerson SN, Ene CI, Levitt MR, Chowdhary MM, Ko AL, Chesnut RM (2018). Timing of cranioplasty: a 10.75-year single-center analysis of 754 patients. J Neurosurg.

[51] Mukherjee S, Thakur B, Haq I, Hettige S, Martin AJAJ (2014). Complications of titanium cranioplasty - a retrospective analysis of 174 patients. Acta Neurochir (Wien).

[52] Nagayama K, Yoshikawa G, Somekawa K, Kohno M, Segawa H, Sano K, Shiokawa Y, Saito I (2002). Cranioplasty using the patient’s autogenous bone preserved by freezing - an examination of post-operative infection rates. No Shinkei Geka.

[53] Oladunjoye AO, Schrot RJ, Zwienenberg-Lee M, Muizelaar JP, Shahlaie K (2013). Decompressive craniectomy using gelatin film and future bone flap replacement. J Neurosurg.

[54] Paredes I, Castaño-León AM, Munarriz PM, Martínez-Perez R, Cepeda S, Sanz R, Alén JF, Lagares A (2015). Cranioplasty after decompressive craniectomy. A prospective series analyzing complications and clinical improvement. Neurocirugia.

[55] Piedra MP, Ragel BT, Dogan A, Coppa ND, Delashaw JB (2013). Timing of cranioplasty after decompressive craniectomy for ischemic or hemorrhagic stroke: Clinical article. J Neurosurg.

[56] Pierson M, Birinyi PV, Bhimireddy S, Coppens JR (2016). Analysis of decompressive craniectomies with subsequent cranioplasties in the presence of collagen matrix dural substitute and polytetrafluoroethylene as an adhesion preventative material. World Neurosurg.

[57] Posti JP, Yli-Olli M, Heiskanen L, Aitasalo KMJ, Rinne J, Vuorinen V, Serlo W, Tenovuo O, Vallittu PK, Piitulainen JM (2018) Cranioplasty after severe traumatic brain injury: effects of trauma and patient recovery on cranioplasty outcome. Front Neurol 9:223–229. 10.3389/fneur.2018.00223 10.3389/fneur.2018.00223 PMC5904383 29695995

[58] Quah BL, Low HL, Wilson MH, Bimpis A, Nga VDW, Lwin S, Zainuddin NH, Wahab NA, Salek MAA (2016). Is there an optimal time for performing cranioplasties? Results from a prospective multinational study. World Neurosurg.

[59] Rashidi A, Sandalcioglu IE, Luchtmann M (2020). Aseptic bone-flap resorption after cranioplasty - incidence and risk factors. PLoS One.

[60] Roberts SAG, Toman E, Belli A, Midwinter MJ (2016). Decompressive craniectomy and cranioplasty: experience and outcomes in deployed UK military personnel. Br J Neurosurg.

[61] Schoekler B, Trummer M (2014). Prediction parameters of bone flap resorption following cranioplasty with autologous bone. Clin Neurol Neurosurg.

[62] Schuss P, Vatter H, Marquardt G, Imöhl L, Ulrich CT, Seifert V, Güresir E (2012). Cranioplasty after decompressive craniectomy: the effect of timing on postoperative complications. J Neurotrauma.

[63] Shibahashi K, Hoda H, Takasu Y, Hanakawa K, Ide T, Hamabe Y (2017). Cranioplasty outcomes and analysis of the factors influencing surgical site infection: a retrospective review of more than 10 years of institutional experience. World Neurosurg.

[64] Shih F-Y, Lin C-C, Wang H-C, Ho J-T, Lin C-H, Lu Y-T, Chen W-F, Tsai M-H (2019). Risk factors for seizures after cranioplasty. Seizure.

[65] Shimizu S, Morikawa A, Kuga Y, Mouri G, Murata T (2002). Cranioplasty using autogenous bone cryopreserved with dimethylsulfoxide (DMSO). No Shinkei Geka.

[66] Sobani ZA, Shamim MS, Qadeer M, Murtaza S, Bari M, Sobani Z, Zafar S, Bilal N, Enam S (2011). Cranioplasty after decompressive craniectomy: an institutional audit and analysis of factors related to complications. Surg Neurol Int.

[67] Stephens FL, Mossop CM, Bell RS, Tigno T, Rosner MK, Kumar A, Moores LE, Armonda RA (2010). Cranioplasty complications following wartime decompressive craniectomy. Neurosurg Focus.

[68] Sundseth J, Sundseth A, Berg-Johnsen J, Sorteberg W, Lindegaard K-F (2014). Cranioplasty with autologous cryopreserved bone after decompressive craniectomy. Complications and risk factors for developing surgical site infection. Acta Neurochir (Wien).

[69] Tantawi D, Armonda R, Valerio I, Kumar AR (2012). Management of decompressive craniectomy defects: modern military treatment strategies. J Craniofac Surg.

[70] Tsang ACO, Hui VKH, Lui WM, Leung GKK (2015). Complications of post-craniectomy cranioplasty: risk factor analysis and implications for treatment planning. J Clin Neurosci.

[71] Wachter D, Reineke K, Behm T, Rohde V (2013). Cranioplasty after decompressive hemicraniectomy: underestimated surgery-associated complications?. Clin Neurol Neurosurg.

[72] Yeap MC, Chen CC, Liu ZH, Hsieh PC, Lee CC, Liu YT, Yi-Chou Wang A, Huang YC, Wei KC, Wu CT, Tu PH (2018). Postcranioplasty seizures following decompressive craniectomy and seizure prophylaxis: a retrospective analysis at a single institution. J Neurosurg.

[73] Honeybul S (2010). Complications of decompressive craniectomy for head injury. J Clin Neurosci.

[74] Honeybul S, Ho KM (2012). How “successful” is calvarial reconstruction using frozen autologous bone?. Plast Reconstr Surg.

[75] Huang Y-H, Yang T-M, Lee T-C, Chen W-F, Yang K-Y (2013). Acute autologous bone flap infection after cranioplasty for postinjury decompressive craniectomy. Injury.

[76] Durham SR, McComb JG, Levy ML, Cohen AR, Menezes AH, Sutton LN (2003). Correction of large (>25 cm2) cranial defects with “reinforced” hydroxyapatite cement: technique and complications. Neurosurgery.

[77] Gilardino MS, Cabiling DS, Bartlett SP (2009). Long-term follow-up experience with carbonated calcium phosphate cement (norian) for cranioplasty in children and adults. Plast Reconstr Surg.

[78] Poetker DM, Pytynia KB, Meyer A, Wackym PA (2004). Complication rate of transtemporal hydroxyapatite cement cranioplasties: a case series review of 76 cranioplasties. Otol Neurotol..

[79] Godil SS, Shamim MS, Enam SA, Qidwai U, Qadeer M, Sobani ZA (2011). Cranial reconstruction after decompressive craniectomy: prediction of complications using fuzzy logic. J Craniofac Surg.

[80] Honeybul S, Ho KM (2011). Long-term complications of decompressive craniectomy for head injury. J Neurotrauma.

[81] Grant GA, Jolley M, Ellenbogen RG, Roberts TS, Gruss JR, Loeser JD (2004). Failure of autologous bone-assisted cranioplasty following decompressive craniectomy in children and adolescents. J Neurosurg.

[82] Stiver SI (2009). Complications of decompressive craniectomy for traumatic brain injury. Neurosurg. Focus.

[83] Bowers CA, Riva-Cambrin JAY, Hertzler DA, Walker ML (2013). Risk factors and rates of bone flap resorption in pediatric patients after decompressive craniectomy for traumatic brain injury. Clinical article. J Neurosurg Pediatr.

[84] Thavarajah D, De Lacy P, Hussien A, Sugar A (2012). The minimum time for cranioplasty insertion from craniectomy is six months to reduce risk of infection-a case series of 82 patients. Br J Neurosurg.

[85] Al-Qattan H, Gernsback JE, Nugent AG, Lyapichev KA, Komotar RJ, Chim H (2017). Heterotopic intracranial skin presenting as chronic draining sinus after remote craniotomy. World Neurosurg.

[86] Kurland DB, Khaladj-Ghom A, Stokum JAJA, Carusillo B, Karimy JK, Gerzanich V, Sahuquillo J, Simard JM (2015). Complications associated with decompressive craniectomy: a systematic review. Neurocrit Care.

[87] Yadla S, Campbell PG, Chitale R, Maltenfort MG, Jabbour P, Sharan AD (2011). Effect of early surgery, material, and method of flap preservation on cranioplasty infections: a systematic review. Neurosurgery.

[88] Spencer R, Manivannan S, Sharouf F, Bhatti MI, Zaben M (2019). Risk factors for the development of seizures after cranioplasty in patients that sustained traumatic brain injury: a systematic review. Seizure.

[89] Gooch MR, Gin GE, Kenning TJ, German JW, Reid Gooch M, Gin GE, Kenning TJ, German JW (2009). Complications of cranioplasty following decompressive craniectomy: analysis of 62 cases. Neurosurg Focus.

[90] Walcott BP, Kuklina EV, Nahed BV, George MG, Kahle KT, Simard JM, Asaad WF, Coumans JVCE (2011). Craniectomy for malignant cerebral infarction: prevalence and outcomes in US hospitals. PLoS One.

