# Supplementary figures and images for: Complications of cranioplasty in relationship to traumatic brain injury: a systematic review and meta-analysis
Source: Neurosurg Rev. 2021 Mar 8;44(6):3125–42. doi: 10.1007/s10143-021-01511-7 (PMC8592959; doi:10.1007/s10143-021-01511-7)

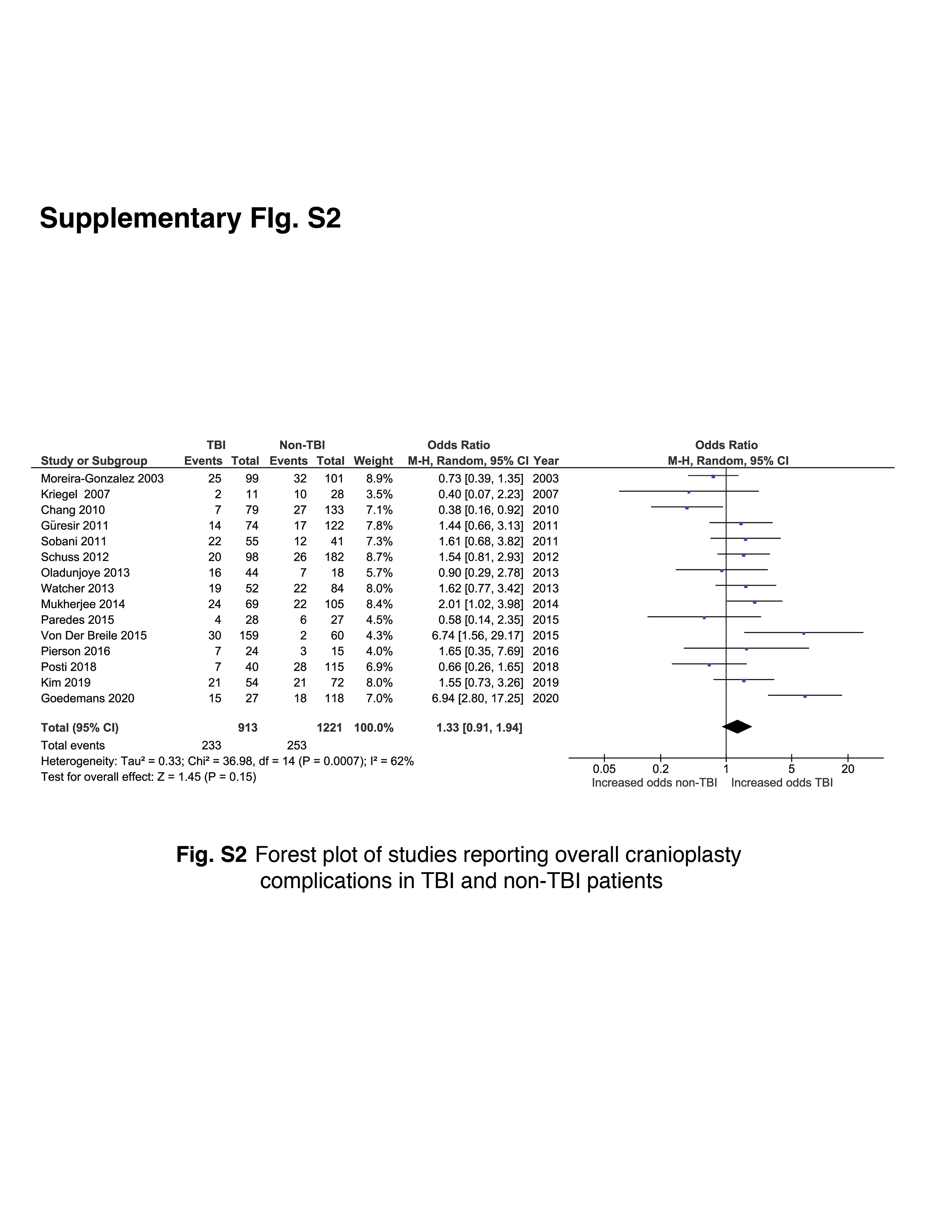

Supplement: Supplementary file 1 — (PNG 584 kb) [file 10143_2021_1511_Fig9_ESM.png]

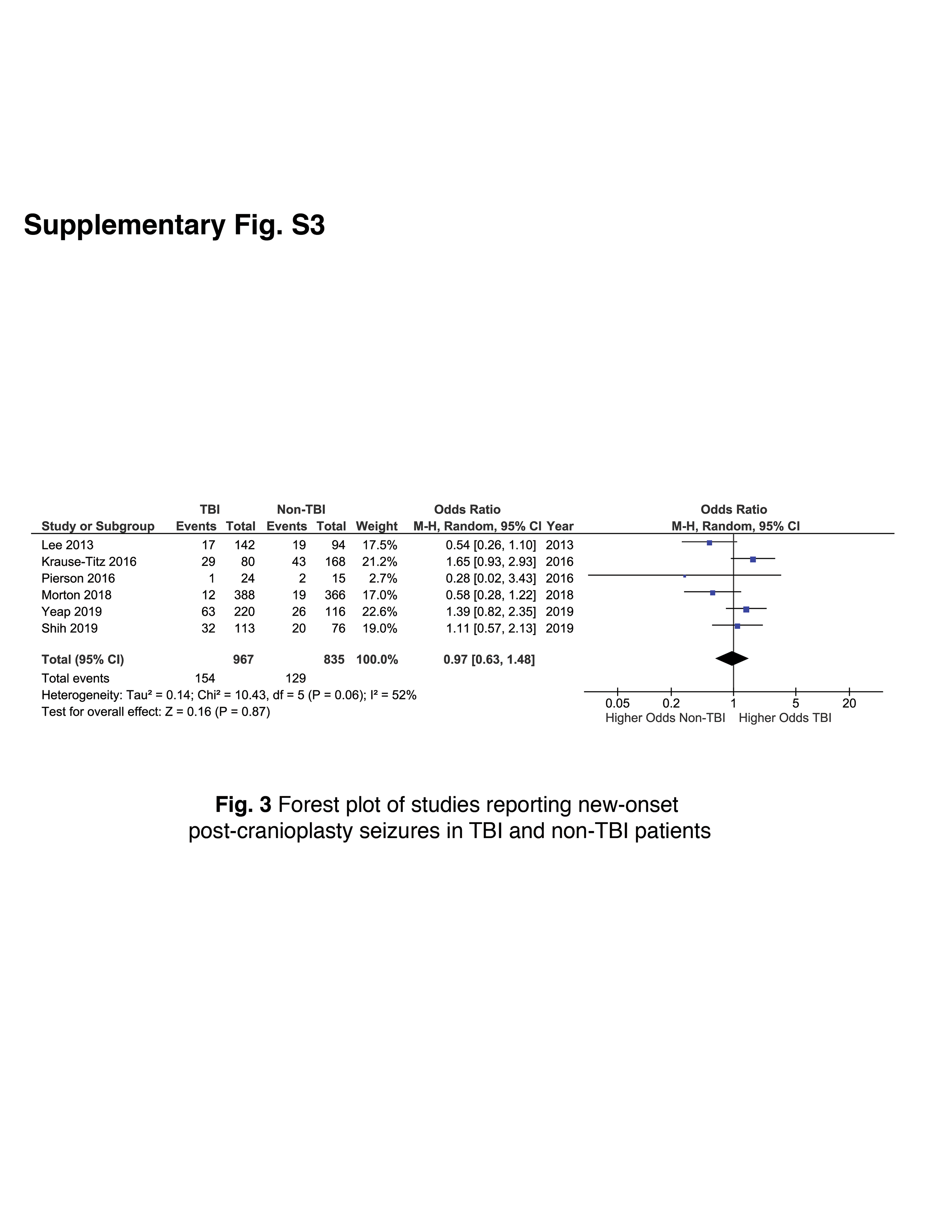

Supplement: Supplementary file 3 — (PNG 415 kb) [file 10143_2021_1511_Fig10_ESM.png]

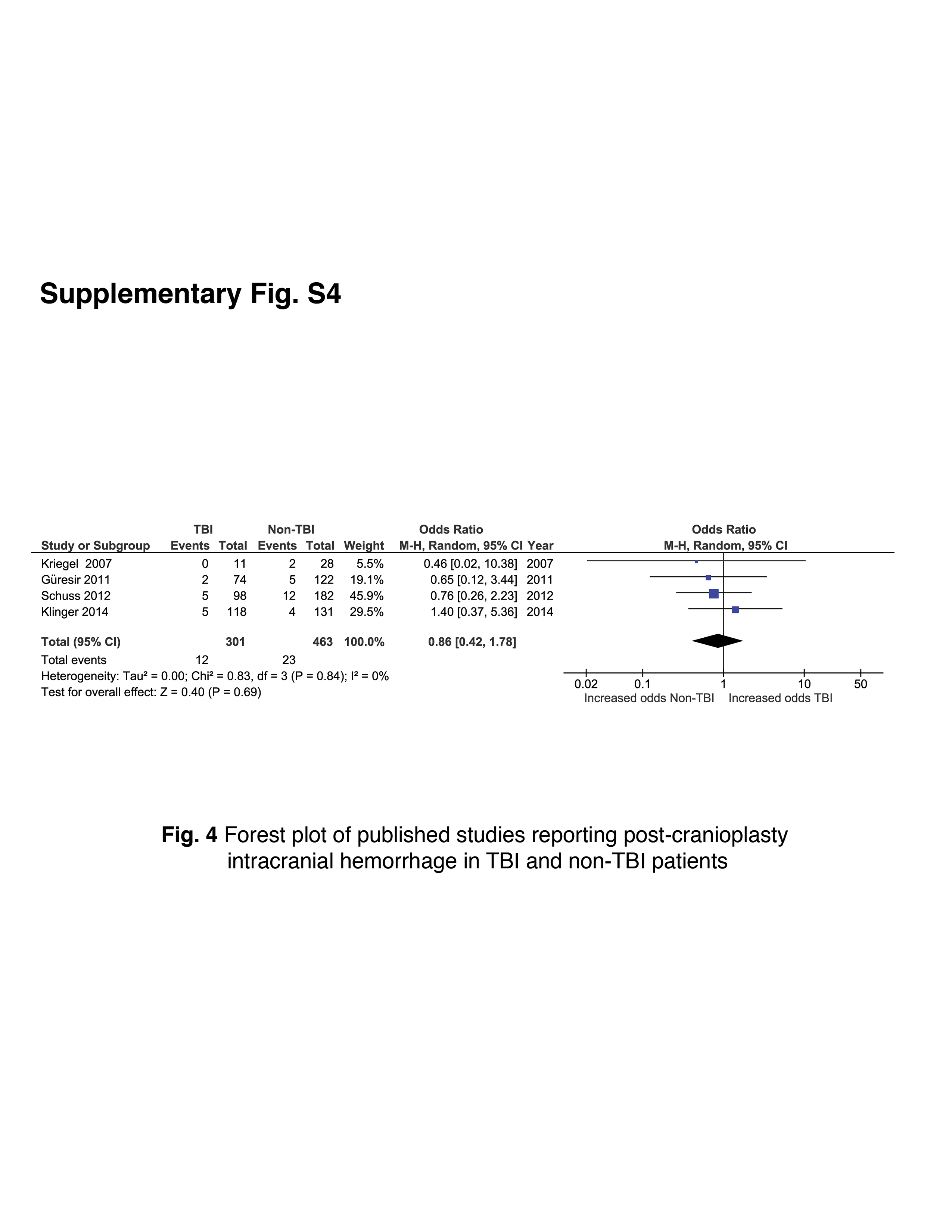

Supplement: Supplementary file 5 — (PNG 372 kb) [file 10143_2021_1511_Fig11_ESM.png]

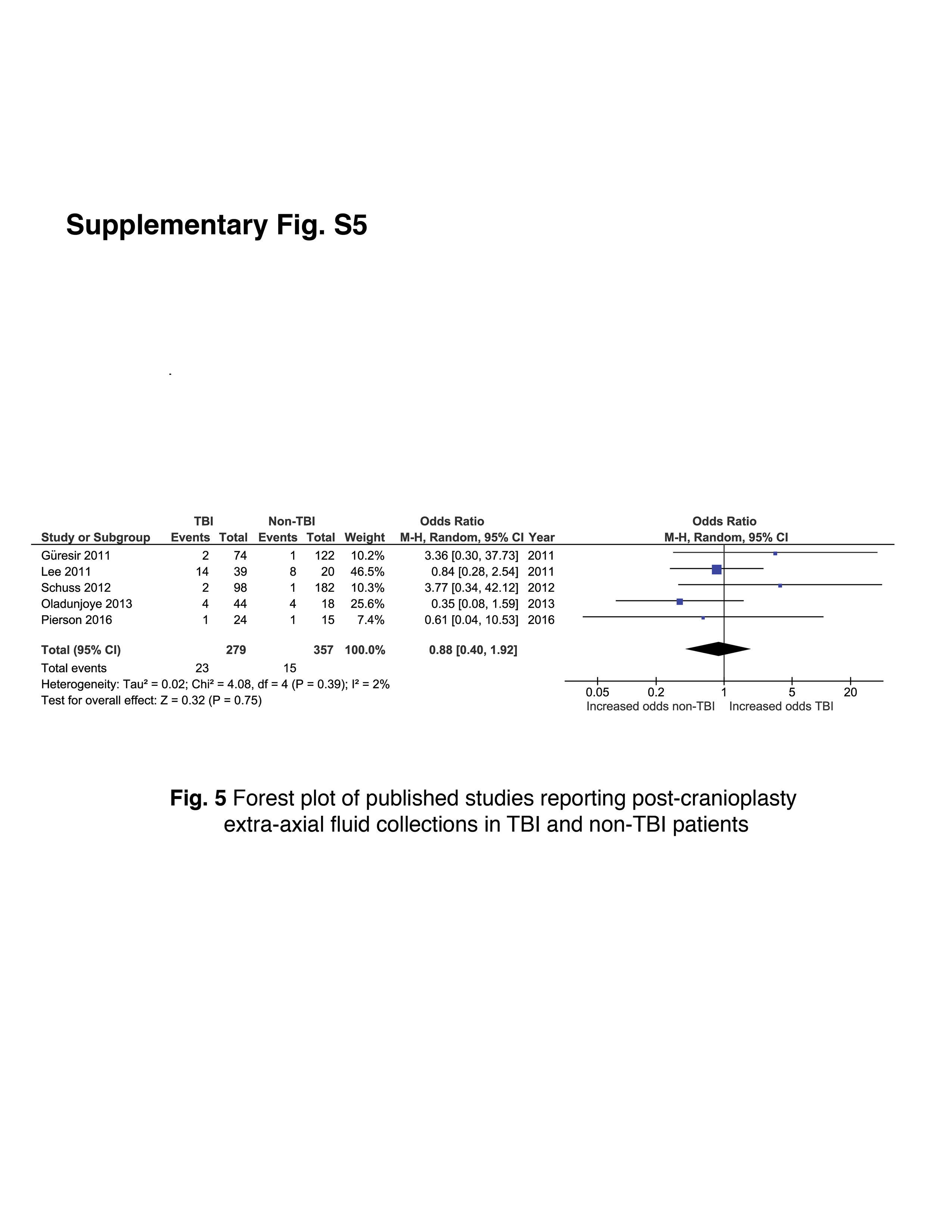

Supplement: Supplementary file 7 — (PNG 390 kb) [file 10143_2021_1511_Fig12_ESM.png]

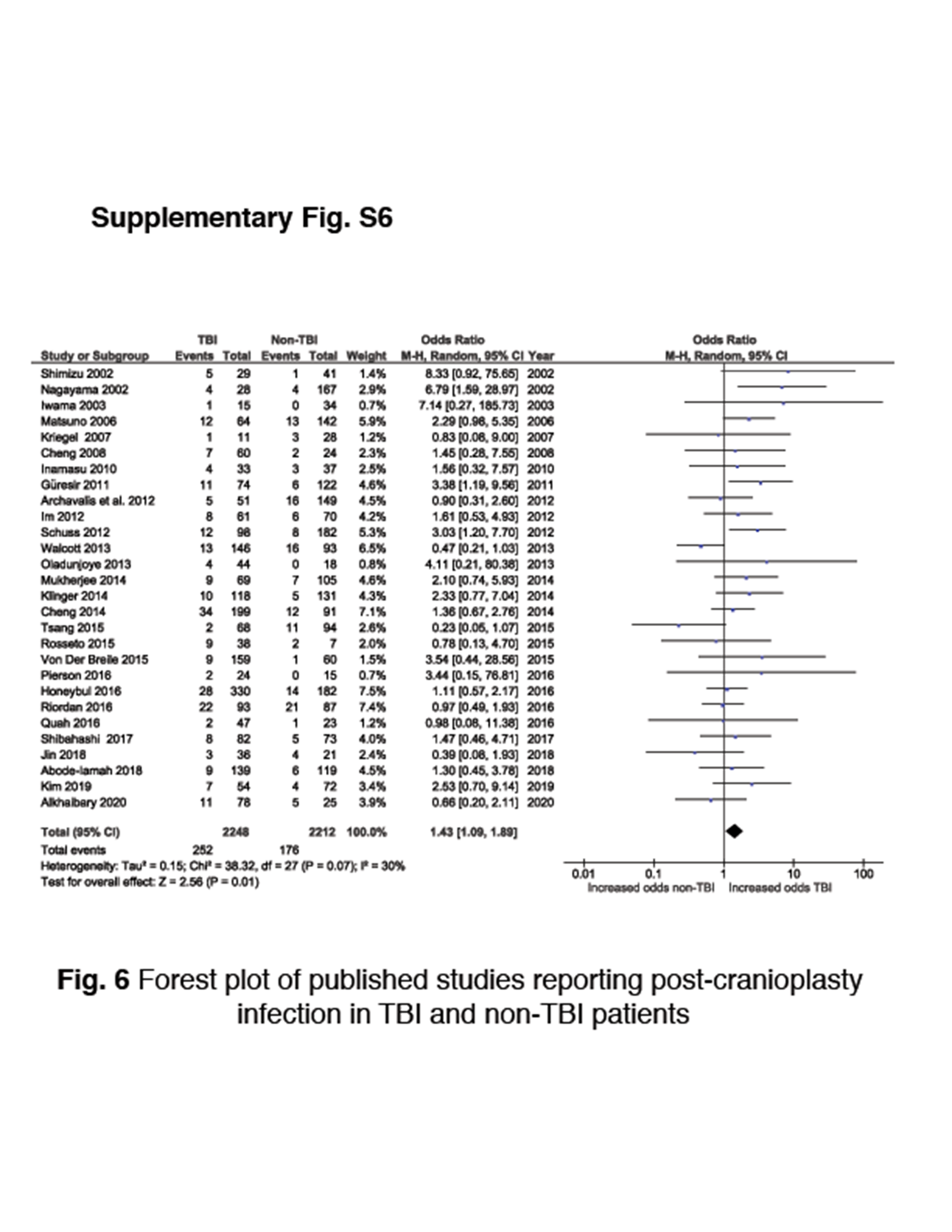

Supplement: Supplementary file 9 — (PNG 1639 kb) [file 10143_2021_1511_Fig13_ESM.png]

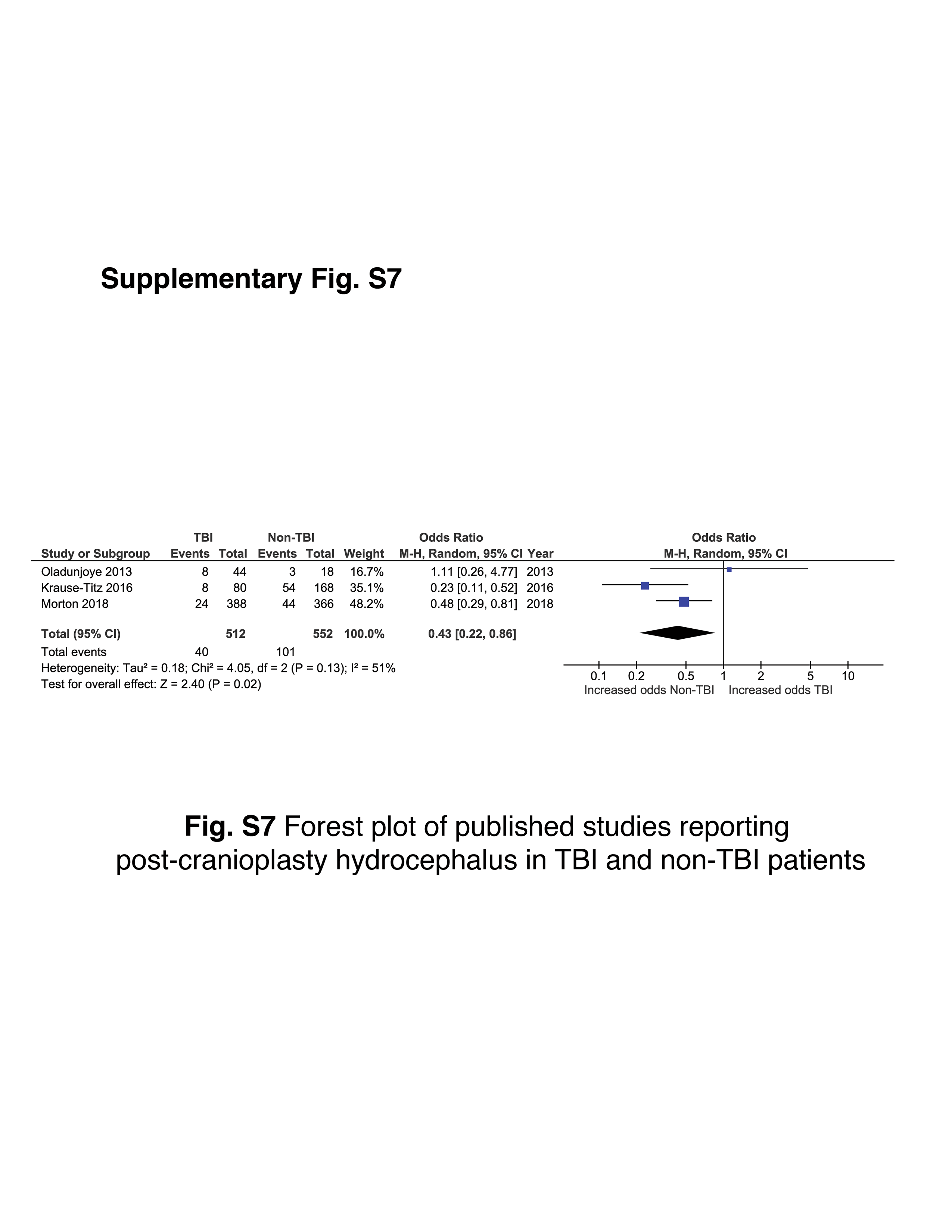

Supplement: Supplementary file 11 — (PNG 370 kb) [file 10143_2021_1511_Fig14_ESM.png]

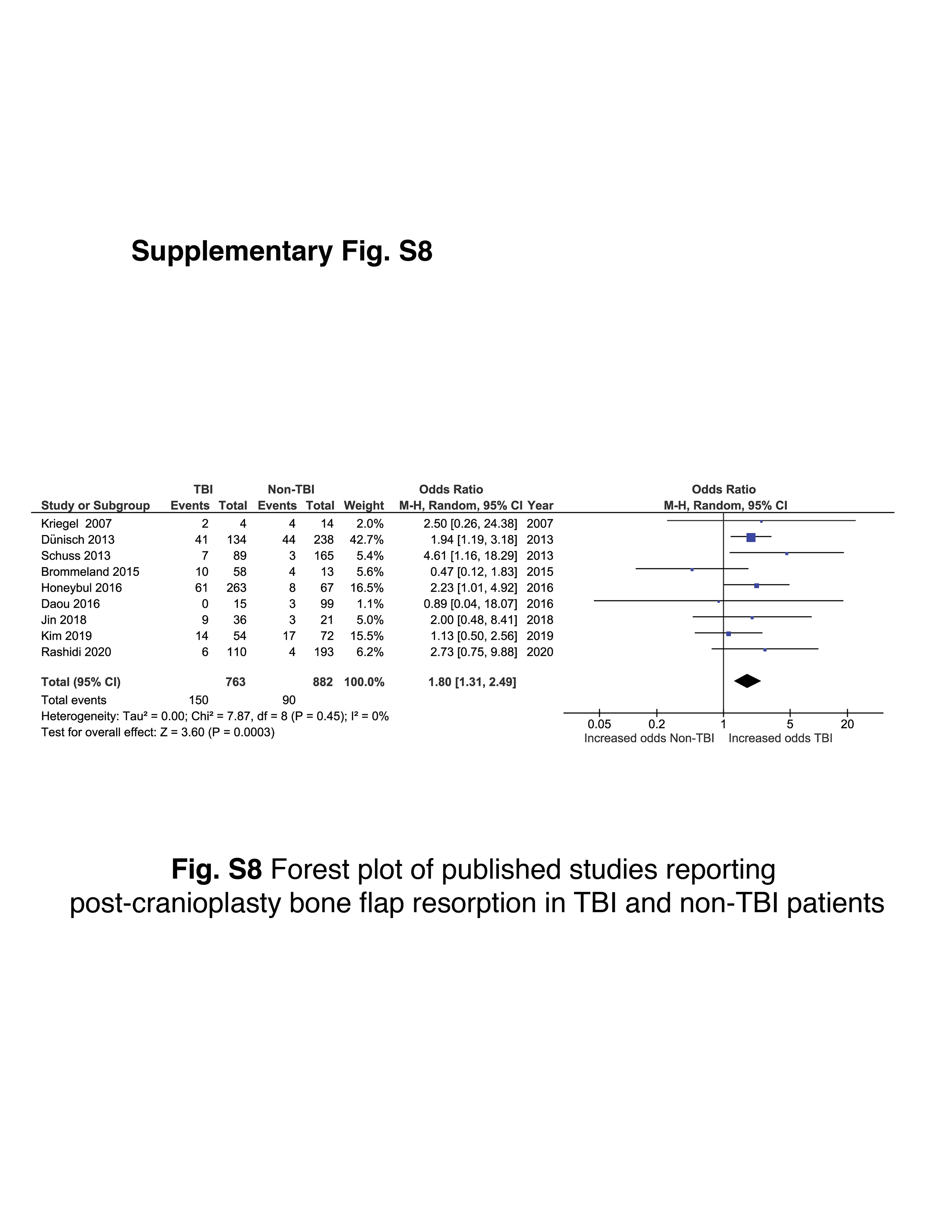

Supplement: Supplementary file 13 — (PNG 489 kb) [file 10143_2021_1511_Fig15_ESM.png]
